# Verification of a Motion Sensor for Evaluating Physical Activity in COPD Patients

**DOI:** 10.1155/2018/8343705

**Published:** 2018-04-23

**Authors:** Seiko Miyamoto, Yoshiaki Minakata, Yuichiro Azuma, Kazumi Kawabe, Hideya Ono, Ryuta Yanagimoto, Tadatoshi Suruda

**Affiliations:** Department of Clinical Laboratory, National Hospital Organization Wakayama Hospital, 1138 Wada Mihama-cho, Hidaka-gun, Wakayama 644-0044, Japan

## Abstract

**Background:**

Objective evaluation of the physical activity (PA) in patients with chronic obstructive pulmonary disease (COPD) is important. We validated a triaxial accelerometer, Active Style Pro HJA-750C® (HJA), and evaluated the necessary conditions for obtaining reproducible data.

**Methods:**

The PA measured by HJA was compared with that measured by two already validated accelerometers in 11 patients with COPD (age: 76.6 ± 6.9, FEV1% predicted: 57.6 ± 18.6). Then, the influence of weather and holidays on the PA and the required number of days to obtain repeatability were examined in 21 patients with COPD (age: 73.0 ± 8.0, FEV1% predicted: 58.7 ± 19.0).

**Results:**

The PA values measured by HJA and those by DynaPort Move Monitor® (DMM) or Actimarker® (AM) were significantly correlated at all intensities (*p*=0.024 at ≥4.0 METs by DMM and *p* < 0.0001 at the rest) except at ≥4.0 METs by AM, though the values measured by HJA were higher than those by AM which was reported to underestimate PA. The durations of PA on rainy days were significantly shorter than those on nonrainy days, but those on holidays were not different from those on weekdays. The values of ICC for 3, 4, or 5 days were higher than 0.8 at all intensities. The PA measured by HJA was correlated with the dyspnea scale FVC and age and tended to correlate with FEV1.

**Conclusions:**

The HJA was validated for evaluating the PA in patients with COPD. This trial is registered with UMIN000016363.

## 1. Introduction

Physical activity (PA) in patients with chronic obstructive pulmonary disease (COPD) is an important issue because it is related to the decline of lung function [1], hospitalizations [2], and mortality [3]. Furthermore, the PA level evaluated by an accelerometer is the strongest predictor of all-cause mortality in COPD [4].

The reports of PA in patients with COPD measured by accelerometers have rapidly increased [5–7]. Recently, the associations between the intensity of PA in COPD and several factors including dyspnea [8], limitation of tidal volume expansion [9], and leg muscle oxygen availability [10] have been reported. DynaPort Activity Monitor® (McRoberts BV, The Hague, Netherlands), its upgraded version, DynaPort Move Monitor (DMM; McRoberts BV, The Hague, Netherlands) [11–14], and SenseWear Armband® (BodyMedia, Pittsburgh, USA) [15–17] are frequently used in Western countries, but are not available in Japan. A compact-sized triaxial accelerometer, the Actimarker (AM; Panasonic, Osaka, Japan), had been available [8, 18–21], but production of the AM ended several years ago. An Activity Monitoring and Evaluation System (Solid Brains Inc., Kumamoto, Japan) is used for COPD [22, 23], but 2 accelerometers are required, and it is rather heavy and relatively complex to use for physicians. Recently, a new compact triaxial accelerometer, Active Style Pro HJA-750C (HJA; Omron Healthcare Co., Ltd, Kyoto, Japan), became available in Japan. Its metabolic equivalent (MET) value is calculated from three different linear regression formulas (sedentary, household, and locomotion) [24, 25]. However, the validity of HJA for patients with COPD has not been evaluated. Furthermore, when the PA is evaluated by HJA, the reproducibility of the data is very important, especially for evaluating the effects of medical intervention on PA.

The main hypothesis in this study was that the HJA is valid for evaluating the PA in patients with COPD. To prove this hypothesis, we tested the reproducibility of HJA compared to that of DMM or AM, which had been validated for patients with COPD. Then, in order to obtain repeatable data, we investigated the influence of weather or holidays on the duration of PA and also the minimal number of days required for analysis. Furthermore, we evaluated the relationships between PA and several demographic factors.

## 2. Materials and Methods

### 2.1. Subjects

For study 1, patients with stable COPD aged 40 or older were recruited from the National Hospital Organization Wakayama Hospital from April to September 2015. For studies 2 and 3, outpatients with stable COPD without any other diseases that might obviously interfere with walking were recruited from the same institute from May 2015 to January 2016 and from November 2016 to October 2017. COPD was defined as postbronchodilator forced expiratory volume in one second (FEV1)/forced vital capacity (FVC) < 0.7. The participants had not been diagnosed with asthma or bronchiectasis [26]. This study was conducted in accordance with the Declaration of Helsinki and was approved by the local ethics committee (IRB Committee of National Hospital Organization Wakayama Hospital; approval number: 1 and 8; approval date: October 31, 2014, and March 20, 2015, resp.).Written informed consent was obtained from all participants.

### 2.2. Design

#### 2.2.1. Study 1

In order to evaluate the validity of the device, HJA compared to DMM or AM, patients attached three devices at the same time in the daytime except while bathing for 7 days (Supplementary S1). When the total measurement duration was less than 8 hours, the data were considered invalid [27, 28]. The data from the first and last days were excluded because the patients went to the hospital for the setup or removal of the devices and were recorded only part of the day [19, 29]. The data from the first 3 valid days include holidays per each patient employed for analysis because the minimal required number of days for evaluated PA in COPD was reported to be 2 to 7 [11, 18, 28, 30–32], and the data from at least 3 days could be obtained from all participants when the invalid days were excluded from measured 7 days (Supplementary S2). Then, the durations of PA according to the intensity (≥2.0 METs, ≥3.0 METs, and ≥4.0 METs) were compared. For DMM, the duration of locomotion (walking + cycling) was employed as an index of the PA.

#### 2.2.2. Study 2

To investigate the influence of the weather or holiday on the duration of PA and determine the minimal required number of days for analysis, the HJA was attached throughout the day except while bathing for 2 weeks. The description of a diary in relation to weather and the kinds of activities was required during the measurement of PA. During the 2 weeks, the data of the first and last days were excluded [19, 29]. When the total measurement duration was less than 8 hours [27, 28] or when unusual activities (e.g., traveling, etc.) were performed, the data were excluded as invalid. The durations of PA on rainy days were compared with those on nonrainy days, and those on holidays (Saturday, Sunday, and legal holidays) were compared with those on weekdays. To determine how many days of measurement are necessary to ensure the repeatability of the measurements, the intraclass correlation coefficient (ICC) values of the duration of PA from the first 2, 3, 4, and 5 days among all evaluable days were evaluated (Supplementary S3).

#### 2.2.3. Study 3

To evaluate the influenced factor for PA, the values of pulmonary function tests and other demographic factors were compared with the values of PA among the patients of study 2. The values of PA were calculated as mean values from the data of the first three nonrainy days except the first and last days (Supplementary S3).

### 2.3. Measurement of PA

HJA, AM, and DMM are all triaxial accelerometers that are compact and worn only at the waist. HJA and AM can record the duration of PA according to the intensity, and DMM can record the time spent in inactive (lying and sitting), static (standing and shuffling), moving (walking, stair walking, and cycling), and not worn.

HJA is a small (40 mm × 52 mm × 12 mm) and lightweight (23.0 g) accelerometer that can continuously monitor the PA for 45 days. The HJA collects the data of triaxial acceleration at a rate of 32 Hz to 12-bit accuracy. The range of the acceleration data of each axis is ±6 G, resulting in a resolution of 3 mG. The acceleration signals, calculated as the average of the absolute values of the accelerometer output of each axis from 10-second epochs at the middle of each activity, were processed for various acceleration output variables. The MET value is calculated from three different linear regression formulas (sedentary, household, and locomotion) produced by the relationship between the value of acceleration and the MET value measured by indirect calorimetry [24, 25].

## 3. Statistical Analysis

Statistical analysis was performed using GraphPad Prism 5 (GraphPad Software, San Diego, CA) and IBM SPSS Statistics (IBM Japan, Tokyo, Japan). The relationships between the PA evaluated by HJA and DMM or AM were assessed by Spearman's rank correlation, and those between HJA and AM were assessed by Bland–Altman plots. The influences of rainy days and holidays were assessed by the Wilcoxon signed-rank test. The repeatability of the data among measured days was assessed by ICC, in which ICC ≥ 0.8 is a generally accepted value for multiple ICCs in accelerometer studies [31–34]. The relationships between the PA and demographic factors were assessed by Spearman's rank correlation and the Kruskal–Wallis test. The significance was considered as less than 0.05.

## 4. Results

### 4.1. Study 1

**Figure 1:**
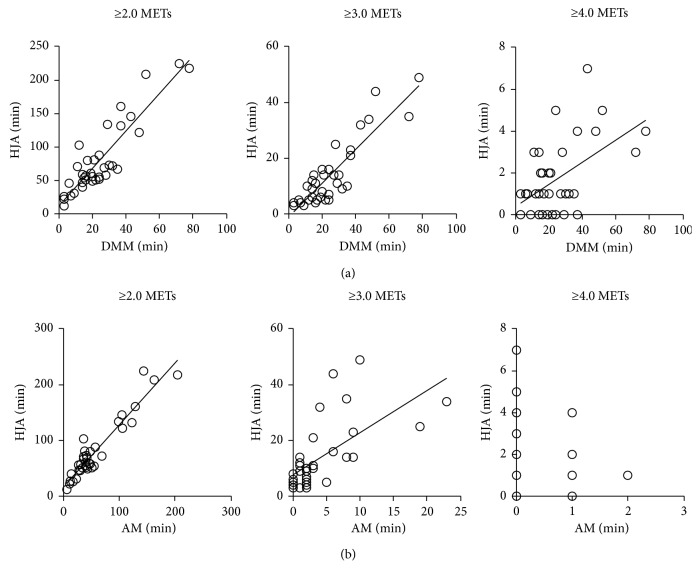
Correlations between the durations of PA measured by HJA and those by DMM and AM. (a) Comparison between HJA and DMM; (b) comparison between HJA and AM. The variables measured by HJA and AM were the duration of PA at ≥2.0 METs, ≥3.0 METs, or ≥4.0 METs. The variables measured by DMM were the duration of locomotion. METs: metabolic equivalents; HJA: Active Style Pro HJA-750C; AM: Actimarker; DMM: DynaPort Move Monitor.

Eleven outpatients with COPD aged 76.6 ± 6.9 were recruited (Table 1). Correlations between the PA measured by HJA and DMM were statistically significant for all indices of PA including duration at ≥2.0 METs and duration of locomotion (*r*=0.808, *p* < 0.0001), ≥3.0 METs and locomotion (*r*=0.801, *p* < 0.0001), and ≥4.0 METs and locomotion (*r*=0.376, *p*=0.024) (Figure 1(a)). Correlations between the PA measured by HJA and AM were also statistically significant for the duration at ≥2.0 METs (*r*=0.832, *p* < 0.0001) and ≥3.0 METs (*r*=0.718, *p* < 0.0001) but not at ≥4.0 METs (*r*=−0.045, *p*=0.795) (Figure 1(b)). The durations at ≥4.0 METs measured by HJA were 0 min in 10 of 36 cases, but those measured by AM were 28 of 36 cases. There were fixed biases between the values of PA measured by HJA and those by AM at all intensities, when a Bland–Altman plot was applied (at ≥2.0 METs: average 95% confidence interval (95% CI) 29.1, 17.1 and limit of agreement (LOA) −11.5, 57.7; at ≥3.0 METs: 95% CI 14.7, 4.8 and LOA −8.61, 28.17; at ≥4.0 METs: 95% CI 2.07, 0.81 and LOA −2.23, 5.12). Most of the values of PA by HJA were higher than those by AM.

### 4.2. Study 2

**Figure 2:**
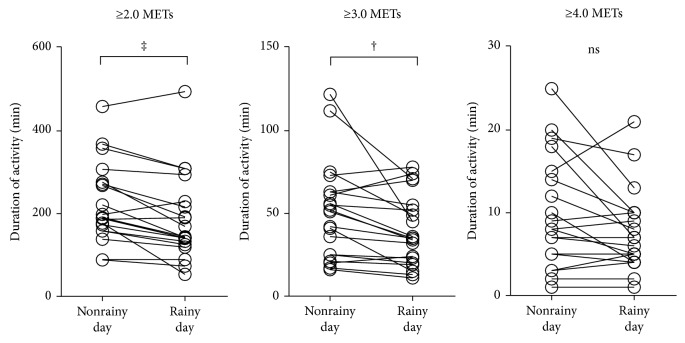
Effect of rainy days on the duration of PA. METs: metabolic equivalents; PA: physical activity; ‡: *p* < 0.001; †: *p* < 0.01.

**Figure 3:**
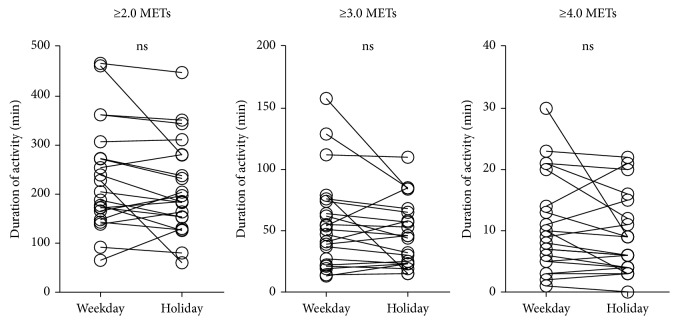
Effect of holidays on the duration of PA. METs: metabolic equivalents; PA: physical activity.

**Figure 4:**
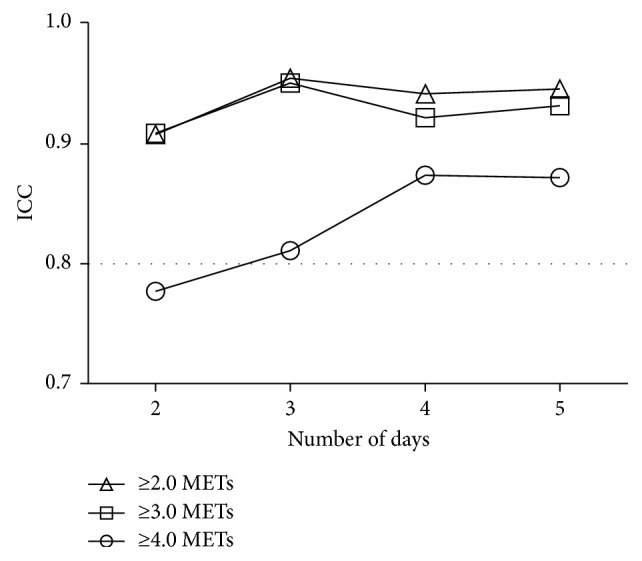
Repeatability of the measurement. Triangle indicates the duration at ≥2.0 METs; square indicates the duration at ≥3.0 METs; circle indicates the duration at ≥4.0 METs. METs: metabolic equivalents; ICC: intraclass correlation coefficient.

Twenty-one outpatients with COPD aged 73.0 ± 8.0 were recruited (Table 1). In one patient, there was no rainy weather during the measurement period, so the effect of rainy days on PA was evaluated with the data of 20 patients. The number of rainy days was 2.6 ± 1.3, nonrainy days 10.0 ± 1.8, holidays 4.0 ± 0.9, and weekdays 8.3 ± 1.2. The durations of PA on rainy days were significantly shorter than those on nonrainy days at ≥2.0 METs (*p* < 0.001) and ≥3.0 METs (*p* < 0.01) but not at ≥4.0 METs (*P*=0.062) (Figure 2). The durations of PA on holidays were not significantly different from those on weekdays (≥2.0 METs, *p*=0.165; ≥3.0 METs, *p*=0.121; and ≥4.0 METs, *p*=0.096) (Figure 3). The values of ICC for 2 days were less than 0.8 at ≥4.0 METs, but those for 3, 4, or 5 days were more than 0.8 at all intensities (Figure 4).

### 4.3. Study 3

The durations of PA were positively correlated with FVC% predicted and negatively with modified Medical Research Council (mMRC) dyspnea scale score and age and tended to be positively correlated with FEV1% predicted (Table 2).

## 5. Discussion

### 5.1. Overviews

The correlations of PA at ≥2.0, ≥3.0, and ≥4.0 METs measured by HJA were confirmed with those measured by DMM or AM except at ≥4.0 METs by AM. However, most of the values of the duration of PA by HJA were higher than those measured by AM. When the PA of COPD was evaluated by HJA, the duration of PA was significantly shorter on rainy days than that on nonrainy days at ≥2.0 and ≥3.0 METs. In order to obtain reproducibility in the duration of PA measured by HJA, the data of at least 3 nonrainy days were necessary at all intensities. An available new triaxial accelerometer, the HJA, was validated, and the method for selecting the repeatable data was demonstrated in the current study. These results could provide Japanese physicians with the available method for evaluating the PA in patients with COPD.

### 5.2. Validation of HJA with AM and DMM (Study 1)

HJA is a more compact triaxial accelerometer than AM, and the two accelerometers can evaluate the same parameters. DMM is distributed widely mainly in Europe [14] and is one of the recommended devices for evaluating PA in patients with COPD [30]. DMM can evaluate the duration of PA according to the kinds of activities, which are not entirely the same parameters as those for HJA. Both DMM and AM have been used for the measurement of PA in patients with COPD [14, 18]. In the current study, significant correlations were confirmed between HJA and DMM or AM at all intensities except ≥4.0 METs by AM, though the durations of PA at ≥2.0, ≥3.0, and ≥4.0 METs by HJA were compared to the duration of locomotion by DMM.

For the PA at ≥4.0 METs, AM could detect only 28% of cases (10 out of 36), but HJA could detect 74% of cases (28 out of 36). This might be the reason why no correlation was observed between AM and HJA. Furthermore, most of the values of PA measured by AM were also lower than those by HJA at ≥2.0 and ≥3.0 METs, and fixed biases between HJA and AM were observed with Bland–Altman plots. The AM underestimated METs for all nonlocomotive activities except for hanging clothes and for all locomotive activities except for climbing down the stairs compared to the energy expenditure evaluated by the expired gas using the Douglas bag method [35]. The HJA might detect the PA more precisely than AM. On the other hand, the PA measured by DMM can well reflect the activity-related energy expenditure measured by the doubly labelled water indirect-calorimetry method [14].

### 5.3. Conditions for Measurement of PA (Study 2)

As the composition of day-to-day variations in PA is important for evaluating the PA, factors that could affect the values of PA should be minimized. First, inclement weather might be one of the factors that could suppress PA. The PA on rainy days was significantly lower than that on nonrainy days at all intensities. These results were compatible with those of previous reports [18, 36]. Second, holidays might be another factor. The PA in COPD on holidays was not different from that on weekdays at all intensities. Steele et al. showed that the amount of physical activity performed during weekdays or weekend days is similar in retired persons [5], which is compatible with our results. However, Pitta et al. reported that the data of weekend days and holidays should be excluded [11]. Tudor-Locke et al. also reported that Sunday should be the last day to enter the analysis [34]. These differences might be affected by whether the subject is employed or not. In the current study, 6 patients had no occupation and 10 out of 15 patients who had occupations worked on both weekdays and holidays (farmer, forestry, and fisherman). This might be the reason why the PA was not different between holidays and weekdays in this study.

In order to obtain repeatability for the PA results at all intensities (ICC ≥ 0.8), the data of 3 days were required. In most of the previous reports that evaluated the PA in COPD with an accelerometer, the data of 3 to 5 days were required [5, 18, 31]. These reports are compatible with our results. Pitta et al. reported that 2 days were enough, but they evaluated the repeatability with ICC ≥ 0.7, which was less reliable than that of the current report (ICC ≥ 0.8) [11]. These results suggest that, for the purpose of obtaining reproducible data, it would be better to analyze the data from 3 nonrainy days.

### 5.4. Influenced Factor for PA (Study 3)

The durations of PA were negatively correlated with the mMRC grade. PA in COPD was reported to be correlated with mMRC [8, 12, 37], and the cutoff value was mMRC 2 [8]. These findings are compatible with our results. Dyspnea might be a key element in modulating the PA in COPD. The durations of PA were correlated with FVC% predicted and tended to correlate with FEV1% predicted. It was reported that a correlation between pulmonary function and PA was observed but it was weak [7, 12, 38], which was also compatible with our results. The durations of PA at ≥4.0 METs were negatively correlated with age. Older people were more likely to be inactive than younger people [39]. In recommendation from the American College of Sports Medicine and the American Heart Association, the recommended level of PA is lower in older people than that in younger ones [39, 40]. Recently, the associations between the intensity of PA in COPD and limitation of tidal volume expansion [9] or leg muscle oxygen availability [10] have also been reported.

### 5.5. Limitations

There are several limitations that need to be addressed: first, the numbers of recruited subjects in both studies were small. The small number might have influence on the effects of holidays in study 2 and on the correlation with pulmonary function especially FEV1 in study 3. Second, though the effects of weather and holidays were evaluated, seasonal effects were not evaluated. Since the weather or temperature might influence the PA in COPD [36, 41], further study is required to determine such possible effects. Third, comorbidities and psychological conditions were not evaluated in this study. Many factors might have influence on the PA, especially on the results of study 3. Effects of these factors should be evaluated in other studies.

## 6. Conclusions

A new triaxial accelerometer, the HJA, was validated by comparing it with DMM and AM, which had already been validated for measuring the PA in patients with COPD. When the PA in COPD was measured with HJA, the data of 3 nonrainy days were necessary for obtaining reproducible data. The PA was associated with the dyspnea grade and pulmonary function.

## Figures and Tables

**Table 1:** Characteristics.

	Study 1	Study 2
Age (years)	76.6 ± 6.9	73.0 ± 8.0
Gender (M/F) (*n*)	11/1	20/1
Smoking history		
Pack-years	96.1 ± 47.5	56.7 ± 36.7
Curr/ex/non (*n*)	4/7/1	7/13/1
Body mass index	19.9 ± 2.5	20.4 ± 4.0
GOLD stage (I/II/III/IV) (*n*)	1/5/6/0	3/10/7/1
mMRC scale (0/1/2/3/4) (*n*)	1/4/1/6/0	6/9/3/3/0
Pulmonary function		
FVC (L)	2.78 ± 0.60	2.92 ± 0.64
FEV1 (L)	1.43 ± 0.37	1.57 ± 0.52
FEV1/FVC (%)	51.7 ± 9.2	53.1 ± 8.6
FEV1% predicted (%)	57.6 ± 18.6	58.7 ± 19.0
Occupation (yes/no)	8/4	15^∗^/6

^∗^Farmer: 7; forestry: 2; fisherman: 1; driver: 1; carpenter: 1; concrete work: 1; iron industry: 1; clerical: 1; M: male; F: female; curr: current smoker; ex: ex-smoker; non: nonsmoker; GOLD: Global Initiative for Chronic Obstructive Lung Disease; mMRC: modified Medical Research Council; FVC: forced vital capacity; FEV1: forced expiratory volume in one second. Data are presented as means ± SD.

**Table 2:** Correlations between physical activity and demographic factors.

	≥2.0 METs		≥3.0 METs		≥4.0 METs	
	*r* value	*p* value	*r* value	*p* value	*r* value	*p* value
Age	0.061	0.792	−0.293	0.197	−0.437	0.048
BMI	0.024	0.918	0.031	0.895	0.240	0.296
Smoking (pack-year)	−0.170	0.462	0.194	0.399	0.331	0.143
mMRC score	—	0.024	—	0.004	—	0.038
Goddard score	−0.405	0.068	−0.289	0.204	−0.197	0.392
FVC% predicted	0.403	0.070	0.487	0.025	0.448	0.041
FEV1/FVC	0.244	0.286	0.341	0.130	0.349	0.121
FEV1% predicted	0.409	0.066	0.424	0.056	0.381	0.088
IC	−0.335	0.138	−0.055	0.806	0.068	0.769

BMI: body mass index; mMRC: modified Medical Research Council; FVC: forced vital capacity; FEV1: forced expiratory volume in one second; IC: inspiratory capacity.

## Abbreviations

PA: Physical activity
COPD: Chronic obstructive pulmonary disease
DMM: DynaPort Move Monitor
AM: Actimarker
HJA: Active Style Pro HJA-750C
ICC: Intraclass correlation coefficient
METs: Metabolic equivalents.

## Supplementary Materials

Supplementary 1 Figure S1: setup of 3 triaxial accelerometers. HJA: Active Style Pro HJA-750C; AM: Actimarker; DMM: DynaPort Move Monitor.

Supplementary 2 Figure S2: numbers of patients and measurement days of study 1. HJA: Active Style Pro HJA-750C; AM: Actimarker; DMM: DynaPort Move Monitor.

Supplementary 3 Figure S3: numbers of patients and measurement days of studies 2 and 3. HJA: Active Style Pro HJA-750C.
