# Supplementary figures and images for: Verification of a Motion Sensor for Evaluating Physical Activity in COPD Patients
Source: Can Respir J. 2018 Apr 23;2018:8343705. doi: 10.1155/2018/8343705 (PMC5937578; doi:10.1155/2018/8343705)

Supple Figure S1


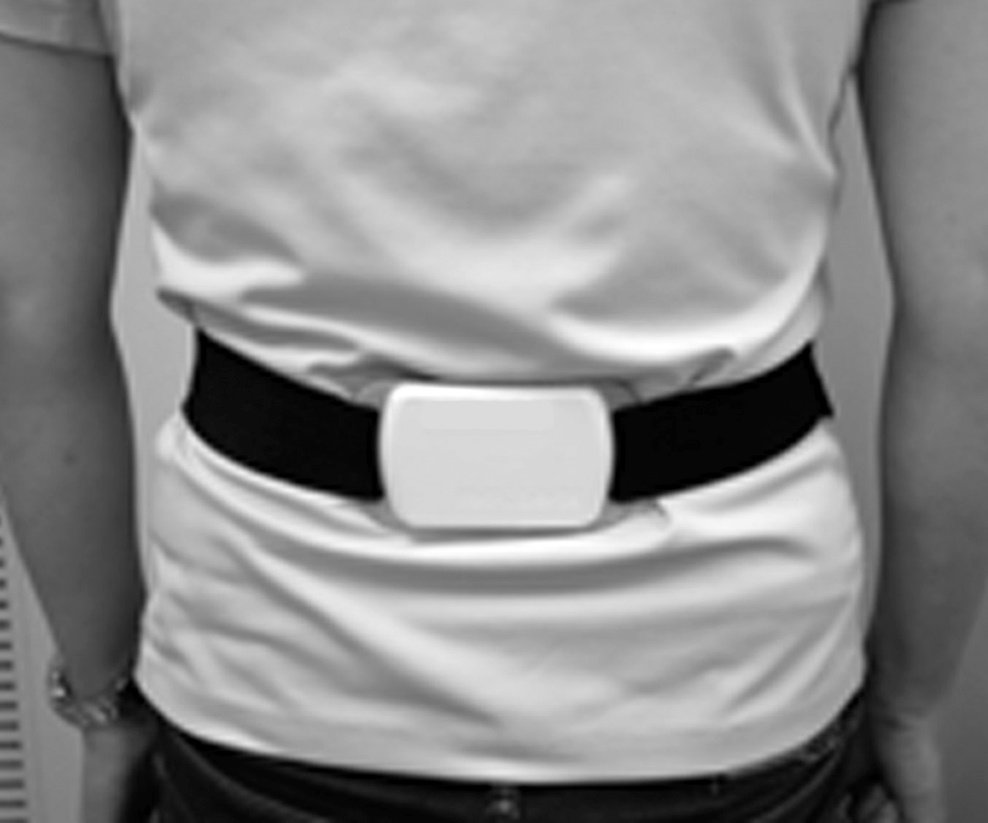

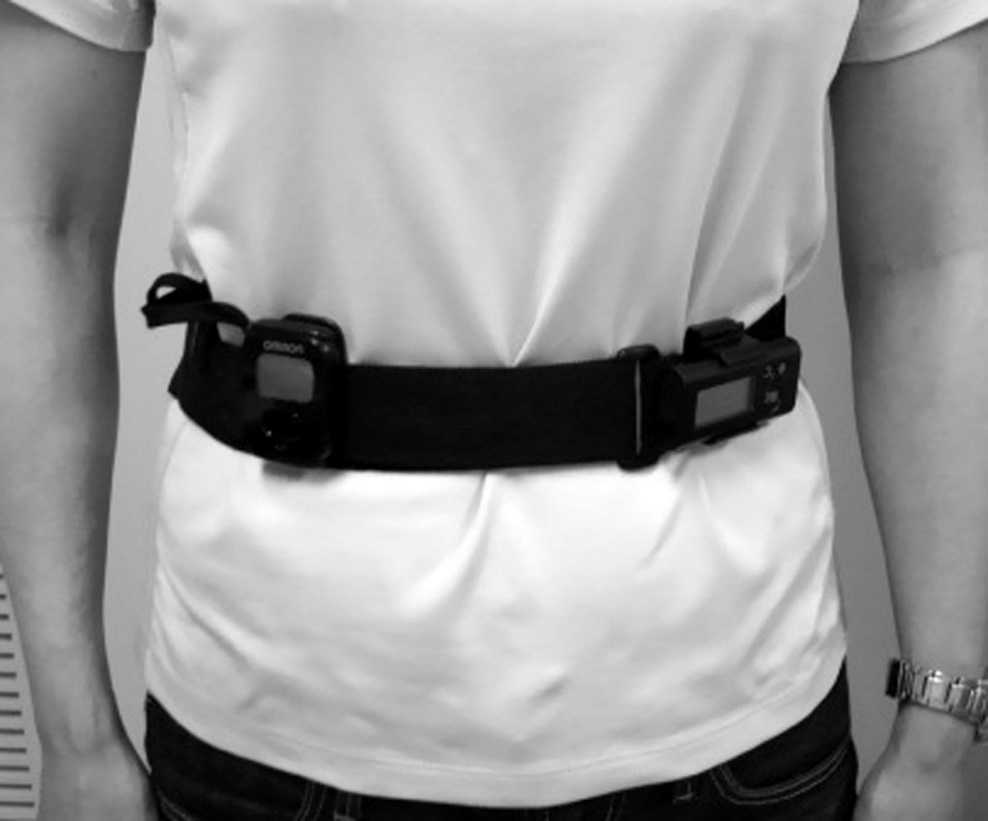


AM

HJA

DMM


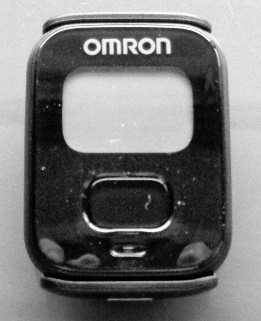


Back


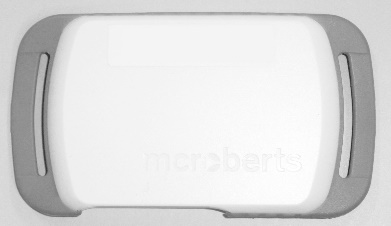

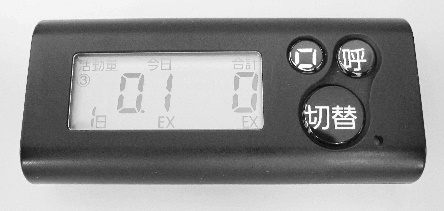


Front

Supplement: Supplementary 1 — Figure S1: setup of 3 triaxial accelerometers. HJA: Active Style Pro HJA-750C; AM: Actimarker; DMM: DynaPort Move Monitor. [file 8343705.f1.docx]
