# Supplementary material for: Verification of a Motion Sensor for Evaluating Physical Activity in COPD Patients
Source: Can Respir J. 2018 Apr 23;2018:8343705. doi: 10.1155/2018/8343705 (PMC5937578; doi:10.1155/2018/8343705)
Supplement: Supplementary 2 — Figure S2: numbers of patients and measurement days of study 1. HJA: Active Style Pro HJA-750C; AM: Actimarker; DMM: DynaPort Move Monitor. [file 8343705.f2.docx]

Suppl Figure S2

33 days excluded due to

- First and last days

12 patients

24 measurement days

- Measured for <8 hrs

9 measurement days

Valid data

12 patients

51 measurement days

15 days excluded due to

- 4th day or more

15 measurement days

Analysis for **Study 1**

12 patients

36 measurement days

Valid data of at least 3 days were obtained from all patients

Enrollment

Worn HJA, AM and DAM for 7 days

12 patients

84 measurement days
