# Supplementary material for: Verification of a Motion Sensor for Evaluating Physical Activity in COPD Patients
Source: Can Respir J. 2018 Apr 23;2018:8343705. doi: 10.1155/2018/8343705 (PMC5937578; doi:10.1155/2018/8343705)
Supplement: Supplementary 3 — Figure S3: numbers of patients and measurement days of studies 2 and 3. HJA: Active Style Pro HJA-750C. [file 8343705.f3.docx]

Suppl Figure S3

56 days excluded due to

- First and last days

21 patients

42 measurement days

- Days measured for <8hrs

10 measurement days

- Unusual activities

4 measurement days

Valid data

21 patients

238 measurement days

1 patient excluded due to

- No rainy day during 2 weeks

1 patient,

10 measurement days

Analysis for **Study 2** (holidays, required number of days)

21 patients

238 measurement days

Analysis for **Study 2** (weather)

20 patients

228 measurement days

Analysis for **Study 3**

Use mean values of 3 days

21 patients

63 measurement days

175 days excluded due to

- Rainy days

52 measurement days

- 4th day or more

123 measurement days

Enrollment

Worn HJA for 14 days

21 patients

294 measurement days
